# Clinical course after corticosteroid therapy in IgG4-related aortitis/periaortitis and periarteritis: a retrospective multicenter study

**DOI:** 10.1186/ar4671

**Published:** 2014-07-23

**Authors:** Ichiro Mizushima, Dai Inoue, Motohisa Yamamoto, Kazunori Yamada, Takako Saeki, Yoshifumi Ubara, Shoko Matsui, Yasufumi Masaki, Takashi Wada, Satomi Kasashima, Kenichi Harada, Hiroki Takahashi, Kenji Notohara, Yasuni Nakanuma, Hisanori Umehara, Masakazu Yamagishi, Mitsuhiro Kawano

**Affiliations:** Division of Rheumatology, Department of Internal Medicine, Kanazawa University Graduate School of Medicine, 13-1 Takara-machi, Kanazawa Ishikawa, 920-8640 Japan; Department of Radiology, Kanazawa University Graduate School of Medical Science, 13-1 Takara-machi, Kanazawa, Ishikawa, 920-8640 Japan; The First Department of Internal Medicine, Sapporo Medical University, South 1, West 16, Chuo-ku, Sapporo, Hokkaido, 060-8543 Japan; Department of Internal Medicine, Nagaoka Red Cross Hospital, 2-297-1 Senshu, Nagaoka, Niigata, 940-2085 Japan; Nephrology Center, Toranomon Hospital, 1-3-1 Kajigaya, Takatsu, Kawasaki, Kanagawa, 212-0015 Japan; Health Administration Center, Sugitani Campus, University of Toyama, 2630 Sugitani, Toyama-shi, Toyama, 930-0194 Japan; Department of Hematology and Immunology, Kanazawa Medical University, 1-1 Daigaku, Uchinada, Kahokugun, Ishikawa, 920-0293 Japan; Division of Nephrology, Department of Laboratory Medicine, Kanazawa University, 13-1 Takara-machi, Kanazawa, Ishikawa, 920-8640 Japan; Department of Pathology and Clinical Laboratory, National Hospital Organization, Kanazawa Medical Center, 1-1 Shimoisibikimachi, Kanazawa, Ishikawa, 920-8650 Japan; Department of Human Pathology, Kanazawa University Graduate School of Medicine, 13-1 Takara-machi, Kanazawa, Ishikawa, 920-8640 Japan; Department of Pathology, Kurashiki Central Hospital, Miwa, Kurashiki, Okayama, 710-8602 Japan; Division of Cardiology, Department of Internal Medicine, Kanazawa University Graduate School of Medicine, 13-1 Takara-machi, Kanazawa, Ishikawa, 920-8640 Japan

## Abstract

**Introduction:**

Immunoglobulin G4 (IgG4)–related aortitis/periaortitis and periarteritis are vascular manifestations of IgG4-related disease. In this disease, the affected aneurysmal lesion has been suspected to be at risk of rupture. In this study, we aimed to clarify the clinical course after corticosteroid therapy in IgG4-related aortitis/periaortitis and periarteritis.

**Methods:**

We retrospectively evaluated clinical features, including laboratory data, imaging findings and the course after corticosteroid therapy, in 40 patients diagnosed with IgG4-related aortitis/periaortitis and periarteritis on the basis of periaortic/periarterial radiological findings, satisfaction of the comprehensive diagnostic criteria or each organ-specific diagnostic criteria, and exclusion of other diseases.

**Results:**

The patients were mainly elderly, with an average age of 66.4 years and with a marked male predominance and extensive other organ involvement. Subjective symptoms were scanty, and only a small proportion had elevated serum C-reactive protein levels. The affected aorta/artery were the abdominal aortas or the iliac arteries in most cases. Thirty-six patients were treated with prednisolone, and the periaortic/periarterial lesions improved in most of them during the follow-up period. Two (50.0%) of four patients with luminal dilatation of the affected lesions before corticosteroid therapy had exacerbations of luminal dilatation after therapy, whereas none of the twenty-six patients without it had a new appearance of luminal dilatation after therapy.

**Conclusions:**

The results of this retrospective multicenter study highlight three important points: (1) the possibility of latent existence and progression of periaortic/periarterial lesions, (2) the efficacy of corticosteroid therapy in preventing new aneurysm formation in patients without luminal dilatation of periaortic/periarterial lesions and (3) the possibility that a small proportion of patients may actually develop luminal dilatation of periaortic/periarterial lesions in IgG4-related aortitis/periaortitis and periarteritis. A larger-scale prospective study is required to confirm the efficacy and safety of corticosteroid therapy in patients with versus those without luminal dilatation and to devise a more useful and safe treatment strategy, including administration of other immunosuppressants.

## Introduction

Immunoglobulin G4 (IgG4)–related disease (IgG4-RD) is a recently recognized systemic inflammatory disease with multiorgan involvement [1–3]. IgG4-RD is characterized by tumefactive lesions, a dense lymphoplasmacytic infiltration with abundant IgG4-positive plasma cells, storiform fibrosis and elevated serum IgG4 levels. Writing from a pathological viewpoint, Stone *et al*. [4] and Kasashima *et al*. [5, 6] described some patients with chronic aortitis/periaortitis or inflammatory aortic aneurysm as also having an IgG4-related condition. Microscopically, these lesions have a predilection for the adventitia and periaortic/periarterial tissue, although they also affect the media, indicating the disease have not just a periaortitis component but also an aortitis one [4–9]. In addition, Inoue *et al*. reported characteristic computed tomography (CT) findings of 17 Japanese patients with IgG4-related periaortitis and/or periarteritis. Macroscopically, this disease represents periaortic or periarterial, circumferential or partial, thickened or masslike lesions with or without aneurysmal change [10]. Presumably, IgG4-related periaortitis and periarteritis may have some overlap with IgG4-related retroperitoneal fibrosis. Inoue *et al*. proposed that this discrimination is dependent on the predominant location of the lesions. They deemed *periaortitis* appropriate to refer to lesions with predominant periaortic and concentric involvement, whereas periureteral or plaquelike lesions should be referred to as *retroperitoneal fibrosis*[10]. In this context, the concept of IgG4-related aortitis/periaortitis and periarteritis (PAo/PA) has been proposed [9]. However, the clinical characteristics and course after corticosteroid therapy in patients with IgG4-related PAo/PA have not been well-clarified. Moreover, although corticosteroid therapy has been suspected to increase the risk of aneurysm formation or rupture [5, 8, 10], the precise incidence of these complications and their timing in the clinical course have not been elucidated.

This state of affairs prompted us to undertake the present study to clarify the clinical characteristics and course after corticosteroid therapy in patients with IgG4-related PAo/PA.

## Methods

### Patients

**Figure 1:**
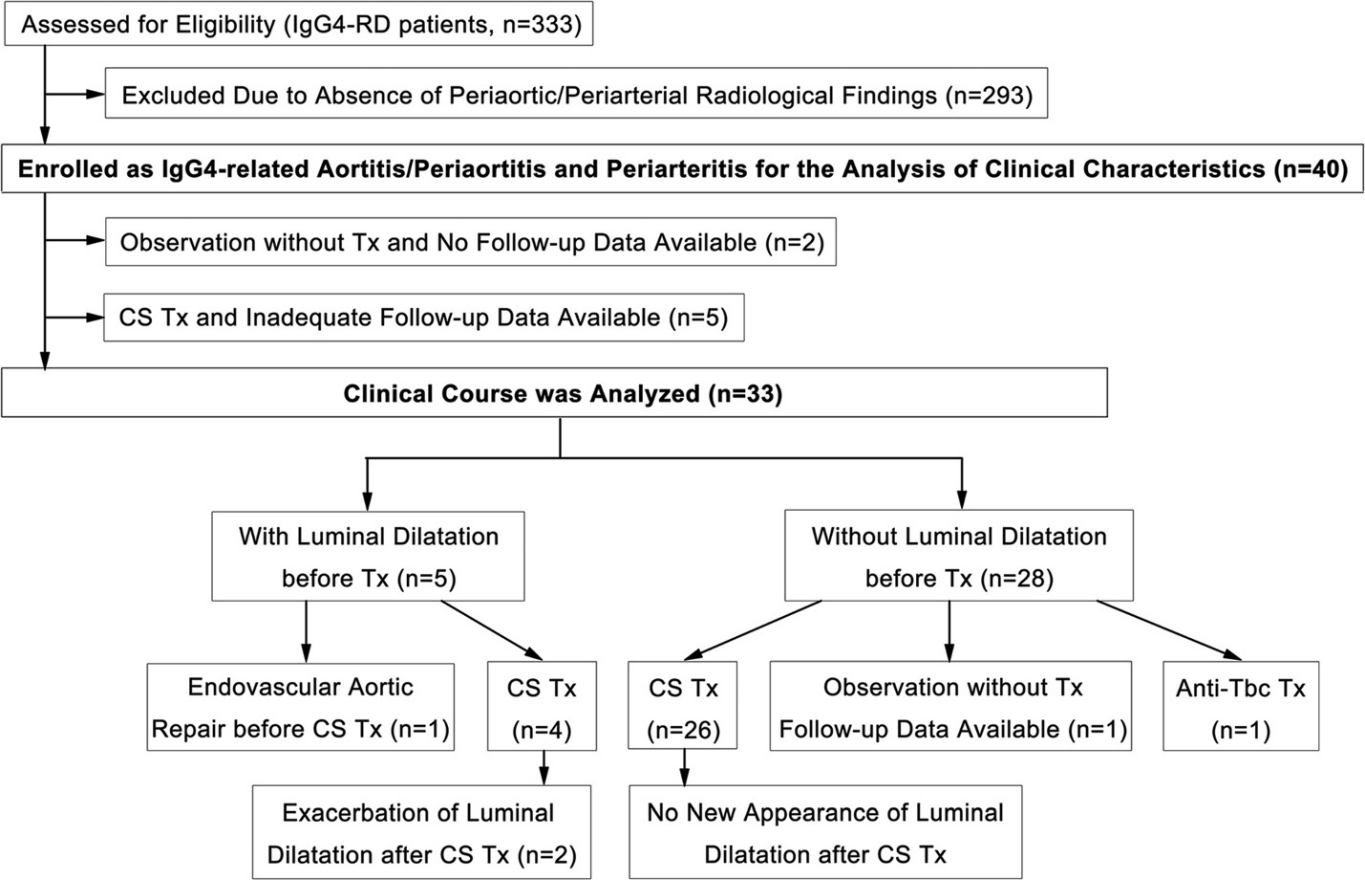
**Flowchart of participants through the study.** CS, corticosteroid; IgG4-RD, IgG4-related disease; Tbc, tuberculosis; Tx, treatment.

From among 333 patients with IgG4-RD at Kanazawa University Hospital, Sapporo Medical University Hospital, Nagaoka Red Cross Hospital, Toranomon Hospital, Toyama University Hospital and Kanazawa Medical University Hospital between 1 January 1995 and 30 September 2013, we identified 40 with IgG4-related PAo/PA (Table 1). The diagnosis of this disease was made on the basis of the presence of consistent periaortic/periarterial radiological findings, the fulfillment of the published comprehensive diagnostic criteria (CDC) [11] or each organ-specific diagnostic criteria [12–14] and exclusion of other diseases. The diagnosis of extravascular lesions was made on the basis of physical examination, imaging findings and/or histopathological examination, in addition to exclusion of other conditions. According to the CDC, 25 patients (patients 2 through 5, 7, 8, 10, 11, 13 through 17, 19, 21 through 23, 26 through 28, 32, 34 through 36 and 38 in Table 1) were diagnosed with definite IgG4-RD, three (patients 1, 9 and 37) with probable IgG4-RD and 12 (patients 6, 12, 18, 20, 24, 25, 29 through 31, 33, 39 and 40) with possible IgG4-RD. Three (patients 6, 18 and 29) of these twelve patients fulfilled the revised diagnostic criteria for autoimmune pancreatitis (AIP) [13]. Well-experienced physicians of this disease diagnosed the remaining nine patients with IgG4-RD on the basis of a consistent clinical picture with elevated serum IgG4 concentrations and exclusion of other diseases. Twenty-nine (80.6%) of thirty-six patients who had extravascular IgG4-related organ involvement underwent biopsy of affected organs and showed histologically typical light microscopic findings [15] and copious IgG4-positive plasma cell infiltration. Histological evaluation of periaortic/periarterial lesions was performed in only one patient (patient 37) by means of incisional biopsy of the periaortic mass lesions, which did not show any vascular structures but histological findings compatible with IgG4-related retroperitoneal fibrosis. We retrospectively evaluated baseline clinical features, including subjective symptoms, laboratory data and imaging findings, in these 40 patients. Because follow-up data were absent or inadequate for seven patients (patients 1, 2, 21, 33, 37, 39 and 40), we limited the analysis of the clinical course to the remaining thirty-three patients (Figure 1). Two patients (patients 8 and 17) had been included in earlier studies ([16] and [17], respectively).

**Table 1:** **Baseline characteristics of 40 patients with IgG4-related aortitis/periaortitis and periarteritis**

						Location of vascular lesion	Luminal dilatation before Tx			Risk factor of arteriosclerosis	Initial PSL Tx (mg/day)
	Follow-up	IgG	IgG4	IgE	CRP			Extravascular lesions			
Patient	(mo)	(mg/dl)	(mg/dl)	(IU/ml)	(mg/dl)				Symptoms		
1	204	1,894	128	543	6.2	AA	(−)	La, Sa	abdo P, fever	(−)	30
2	120	2,840	693	468	0.1	AA	(−)	Sa	(−)	DM, Sm	30
3	96	5,970	3,100	259	1.56	AA	(−)	La, Sa, Hy, Pa	malaise	DM, HT	40
4	78	2,140	557	266	<0.10	AA, IA	(+)	La, Sa	(−)	DL, Sm	50
5	70	1,500	173	151	0.3	IA, SMA	(−)	Pa, RF	abdo P	DM, HT, DL, Sm	20
6	63	2,970	1,330	419	<0.10	AA	(−)	La, Sa, Pa, Ki, RF	malaise	DM, HT, DL, Sm	40
7	63	2,130	715	253	<0.10	IA	(−)	La, Sa	(−)	DM, DL, Sm	40
8	63	2,731	269	975	0.6	AA	(−)	Sa, RF	(−)	DM, HT, DL, Sm	30
9	57	1,790^b^	105^b^	212^b^	1.5^b^	AA, IA	(+)	Hy, Lu, Pa, Ly	(−)	DL	40
10	54	2,570	1,420	345	0.3	AA, IA	(+)	Pa	(−)	DM, HT	20
11	48	2,950	1,540	7.9	<0.1	AA	(−)	Sa, Bi, Pa, Pr	(−)	DM	30
12	43	1,487	196	447	0.6	AA, IA	(−)	RF	abdo P	DM, HT, DL, Sm	0
13	37	2,563	1,330	283	0.09	AA	(−)	Sa, Ki	pollakiuria	DL, Sm	45
14	35	2,319	734	542	1.19	TA, AA	(−)	Sa, Pa, Ki	(−)	DM, DL	40
15	34	1,458	158	452	0.22	AA	(−)	Sa, Ly	(−)	DM, HT, DL, Sm	30
16	27	2,081	870	1,285	0.0	AA, IA	(−)	Sa, RF, Pr	(−)	DM, HT	20
17	27	1,756	408	513	0.2	AA, IA	(−)	Sa, Pa, Ki	(−)	DM, HT, DL, Sm	20
18	27	1,762	144	24	0.32	AA, IA	(+)	Pa	(−)	DL, Sm	20
19	25	2,024	292	1,400	0.14	AA	(−)	Pa, RF	(−)	DM, HT, Sm	40
20	24	2,262	299	443	<0.05	AA	(−)	(−)	(−)	HT	0
21	24	2,184	236	365	0.3	AA	(+)	La, Sa	fever	DM, HT, DL	30
22	22	3,484	1,896	247	0.0	AA	(−)	La, Sa, Pa, Ki, Ly	(−)	Sm	35
23	15	4,171	2,120	<20	0.32	AA, IA	(−)	La, Sa, Ki	(−)	DM, HT, Sm	40
24	13	1,837	261	687	0.06	IA	(−)	RF	(−)	HT, DL	30
25	13	1,454	196	350	0.13	AA, IA	(−)	(−)	abdo P	Sm	15
26	10	2,213	455	NA	0.56	AA, IA	(−)	Sa, Ki	arthralgia	HT, DL, Sm	40
27	10	3,120	1,020	1,760	1.5	AA, IA, IMA	(−)	La, Sa	hoarseness, fever	Sm	30
28	9	2,936	1,070	17	0.0	TA, AA, IA	(−)	La, Sa, Pa, Ki	thirst	DM, DL, Sm	40
29	9	10,121	2,500	<20	0.37	IA	(+)	Pa, Ki, Ly	malaise	DM	50
30	9	1,200	147	NA	2.94	AA, IA	(−)	(−)	fever, malaise	DM, HT	30
31	8	1,475	210	111	0.3	IA	(−)	Sa	(−)	(−)	40
32	4	2,938	1,520	48	0.1	AA	(−)	La, Sa, Ki, RF	(−)	Sm	30
33	4	1,463	672	216	0.13	AA	(−)	Sa, Pl, Ca	(−)	Sm	0
34	3	2,439	782	703	0.2	AA, IA	(−)	La, Ki, RF	(−)	DM, DL, Sm	20
35	3	2,244	503	311	0.0	AA, IA	(−)	Ki	edema	(−)	30
36	2	1,950	711	737	0.0	AA	(−)	La, Sa, Lu, Ki	(−)	Sm	35
37	2	1,328	106	19	0.28	AA, IA	(+)	RF	(−)	DL	0
38	1	4,420	2,680	174	0.1	IA	(−)	La, Sa, Bi, Pa, Ki, Ne	diarrhea	Sm	50
39	1	2,276	835	<20	0.91	IA	(−)	Sa	(−)	HT	15
40	1	1,600	206	212	0.38	AA	(−)	(−)	abdo P, malaise	Sm	30

*AA,* Abdominal aorta; *abdo,* Abdominal; *Bi,* Bile tract; *Ca,* Pericarditis; *CRP,* C-reactive protein; *DL,* Dyslipidemia; *DM,* Diabetes mellitus; *F,* Female; *HT,* Hypertension; *Hy,* Hypophysitis; *IA,* Iliac artery; *IgE,* Serum immunoglobulin E at diagnosis; *IgG,* Serum immunoglobulin G at diagnosis; *IgG4,* Serum immunoglobulin G4 at diagnosis; *IMA,* Inferior mesenteric artery; *Ki,* IgG4-related kidney disease; *La,* Lacrimal gland, *Lu,* Lung; *Ly,* Lymph node; *M,* Male; M*o,* month; *NA,* Not available; *Ne,* Nerve; *P,* Pain; *Pa,* Pancreas; *Pl,* Pleuritis; *Pr, P*rostate; *PSL,* Prednisolone; *RF,* Retroperitoneal fibrosis; *Sa,* Salivary gland; *Sm,* Past or current smoking; *SMA,* Superior mesenteric artery; TA, Thoracic aorta; *Tx,* Treatment. ^b^Value under corticosteroid therapy.

This study was approved by the Medical Ethics Committee of Kanazawa University, the institutional review board of Sapporo Medical University Hospital, the Ethics Committee of Nagaoka Red Cross Hospital, the institutional review board of Toranomon Hospital, the review board of the University of Toyama and the Research Ethics Committee of Kanazawa Medical University. Informed consent for publication of all data and samples was obtained from each patient. The research was conducted in compliance with the Declaration of Helsinki.

### Imaging evaluation

All patients underwent whole-body CT examinations at the time of the initial diagnosis, and follow-up CT data were available for 33 patients, 31 of whom received corticosteroid therapy. All imaging data were reviewed by a single radiologist with extensive experience in IgG4-RD at Kanazawa University Hospital. Periaortic/periarterial lesions were described as circumferential or partial thickened wall of the affected aortas/arteries with homogeneous enhancement visualized by contrast-enhanced CT. At the time of diagnosis, we also evaluated the findings of 2-[^18^F]-fluoro-2-deoxy-D-glucose positron emission tomography/computed tomography (FDG-PET/CT) for 20 patients and of gallium scintigraphy for 12 patients.

At the time of initial diagnostic CT imaging, after noting the affected site of aortas/arteries and extravascular lesions, we measured the maximum vascular wall thickness and diameter of the lumen in both affected and adjacent sites in each lesion. These two values were then longitudinally evaluated in the 33 patients whose follow-up imaging and clinical course information were available.

Improvement and relapse of periaortic/periarterial lesions during the clinical course were defined as decrease and reincrease of vascular wall thickness, respectively, at the same site as the maximum vascular wall thickness measured at the time of initial diagnosis. Luminal dilatation of periaortic/periarterial lesions was defined as being present when the luminal diameter of the affected site was more than 3 mm larger than that of adjacent normal sites. Exacerbation of luminal dilatation was defined as being present when more than 5-mm expansion of the luminal diameter was observed at the same site as the luminal dilatation detected at the time of initial diagnosis.

### Statistical analysis

Statistical analysis was performed using SPSS version 19 software (IBM SPSS, Chicago, IL, USA). The significance of differences between groups was determined using Mann–Whitney *U* test or Wilcoxon signed-rank test, and the significance of differences in frequencies was analyzed with Fisher’s exact probability test. Data are presented as means ± SD. Significant differences were defined as *P* < 0.05.

## Results

### Baseline characteristics

The baseline clinical characteristics of 40 patients are shown in Table 1. Our patient group was composed of 37 men and 3 women with an average age of 66.4 ± 7.1 years (age range, 44 to 75). One patient (patient 9 in Table 1) had been treated with prednisolone (PSL) at a dose of 5.0 mg/day for type 1 AIP. None of the other 39 patients had been treated with any immunosuppressants, including corticosteroids, before their diagnosis. Thirty-six patients (90.0%) had more than one IgG4-related extravascular lesion (average, 2.3 ± 1.5 organs; range, 0 to 6 organs). Involvement of the salivary gland was observed in 25 patients (62.5%), lacrimal gland in 14 (35.0%), pancreas in 14 (35.0%), kidney in 13 (32.5%), retroperitoneum in 13 (32.5%), prostate in 6 (15.0%), lung in 3 (7.5%) and hepatobiliary tract and hypophysis in 2 each (5.0%). The frequency of subjective symptoms was low (fever, 10.0%; abdominal pain, 12.5%; general malaise, 12.5%). Moreover, five of seven patients with luminal dilatation of the affected lesions at the time of diagnosis complained of no subjective symptoms. With regard to the major risk factors of atherosclerosis, diabetes mellitus (DM) was present at the time of diagnosis in 20 patients (50.0%), hypertension (HT) in 17 (42.5%), dyslipidemia (DL) in 18 (45.0%), current smoking in 9 (22.5%) and past smoking in 15 (37.5%). The mean follow-up period of all 40 patients after diagnosis was 33.9 ± 39.8 months (range, 1 to 204 months).

At diagnosis, 37 (92.5%) of 40 patients showed elevated serum IgG4 levels exceeding 135 mg/dl (average, 815 ± 771 mg/dl; range, 105 to 3,100 mg/dl). Thirty-one (77.5%) of forty patients showed elevated serum IgG levels (average, 2,551 ± 1,543 mg/dl; range, 1,200 to 10,121 mg/dl; normal range, 870 to 1,700 mg/dl). Twenty-three (60.5%) of thirty-eight evaluated patients showed elevated serum IgE levels (average, 403 ± 398 IU/ml; range, 7.9 to 1,760 IU/ml; normal range, <250 IU/ml). Although none of the patients had leukocytosis, 16 (41.0%) of 39 evaluated patients had eosinophilia (eosinophils >5%). Six (15.4%) of thirty-nine evaluated patients had hypocomplementemia. Antinuclear antibodies were positive in 16 (40.0%) of 40 patients and the rheumatoid factor in only 3 (8.1%) of 37 evaluated patients. Myeloperoxidase antineutrophil cytoplasmic antibodies (ANCAs) and proteinase 3 ANCAs were not observed in any of the evaluated patients (21 and 14 patients, respectively). Only six (15.0%) of forty patients had elevated serum C-reactive protein (CRP) level (CRP >1 mg/dl).

### Treatment

The respective attending physicians decided the indications for treatment and the treatment regimen. Thirty-six of forty patients were treated with PSL at an average initial dose of 32.6 ± 9.7 mg/day (range, 15 to 50 mg/day) for the lesions associated with IgG4-RD. Only one patient (patient 1 in Table 1) received cyclophosphamide in addition to PSL. Endovascular aortic repair (EVAR) was performed for the periaortic lesions with marked luminal dilatation before corticosteroid therapy in one patient (patient 18) to prevent rupture. We excluded five patients (patients 1, 2, 21, 39 and 40) from the analysis of the clinical course because their follow-up imaging data were not available. During the clinical course of the other 31 patients, the initial PSL dose was generally continued until 2 to 4 weeks after the start of therapy and then gradually tapered. The PSL dose was tapered to 5 to 10 mg/day by 12 months in 18 (94.7%) of 19 patients whose follow-up period was more than 12 months. The average PSL dose at the last review was 10.0 ± 9.2 mg/day. Because one patient (patient 12) showed strong positivity in the tuberculin skin test and interferon γ release assays, he was treated with antituberculosis therapy only. The other three patients (patients 20, 33 and 37) were observed without any treatment (Figure 1).

### Radiological findings at diagnosis

**Figure 2:**
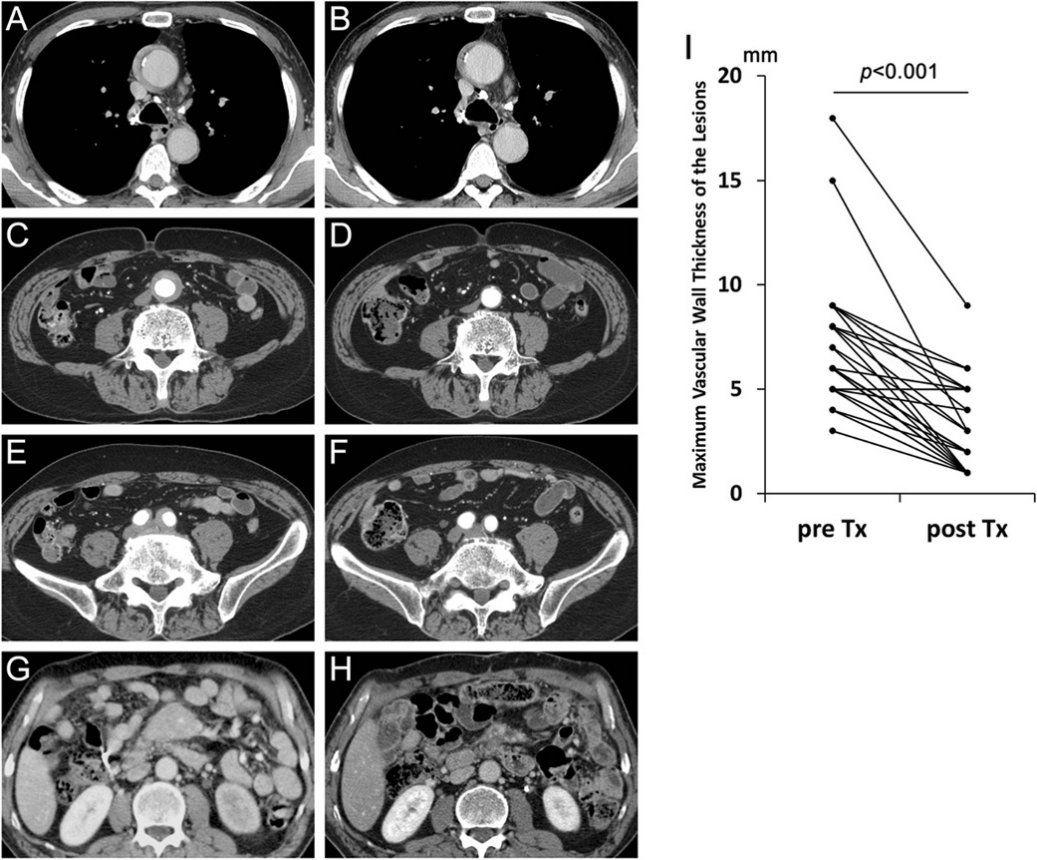
**Contrast-enhanced computed tomography findings of periaortic/periarterial lesions and changes after corticosteroid therapy.** A thoracic aortic lesion **(A)** had slightly improved 1 month after corticosteroid therapy **(B)**, an abdominal aortic lesion **(C)** and an iliac arterial lesion **(E)** had almost disappeared 10 months after therapy (**D** and **F**, respectively) and a superior mesenteric arterial lesion **(G)** showed fair improvement 2 months after therapy **(H)**. Significant pre- to posttherapy decreases in maximum wall thickness of periaortic/periarterial lesions were observed **(I)**. Tx, Treatment.

CT images revealed thickened lesions surrounding the aorta/artery in all patients. The affected aorta/artery data were for two thoracic aortas (Figure 2A), thirty-three abdominal aortas (Figure 2C), twenty-three iliac arteries (Figure 2E), one superior mesenteric artery (Figure 2G) and one inferior mesenteric artery. All 33 abdominal aortic lesions affected the infrarenal abdominal aorta, and only 6 lesions also affected the suprarenal abdominal aorta. CT also revealed typical extravascular lesions, mainly in the salivary glands, lacrimal glands, pancreas and kidney. Sixteen of twenty patients who underwent FDG-PET/CT, and only four of twelve patients who underwent gallium scintigraphy, showed significant uptake of the periaortic/periarterial lesions detected by CT.

### Changes in radiological findings of periaortic/periarterial lesions after corticosteroid therapy

**Figure 3:**
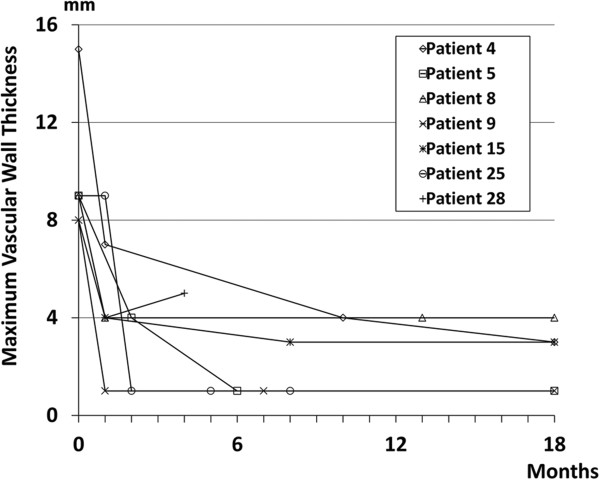
**Changes in maximum vascular wall thickness of periaortic/periarterial lesions after the start of corticosteroid therapy.** Data for patients who underwent follow-up computed tomography within 2 months after the start of therapy are shown.

After corticosteroid therapy, reduction in the thickness of the periaortic/periarterial lesions was observed during an average follow-up period of 30.1 ± 26.2 months (range, 1 to 96 months) in 30 (96.8%) of 31 patients whose clinical course was analyzed (Figure 2), although 1 patient (patient 9 in Table 1) experienced relapse during PSL tapering at a dose of 7.0 mg/day. The average vascular wall thickness of the 34 periaortic/periarterial lesions of 31 patients at the time of diagnosis (7.1 ± 3.0 mm; range, 3 to 18 mm) significantly decreased after corticosteroid therapy (2.7 ± 2.0 mm; range, 1 to 9 mm) (Figure 2I). Generally, obvious radiographic improvement of more than 50% reduction in thickness was observed by 2 months after the start of therapy, after which point some patients showed further improvement and others showed almost no change (Figure 3). Eighteen of the thirty-four lesions had almost completely disappeared by the time of the last review. The rate of improvement, relapse or complete disappearance of the perivascular lesions did not differ significantly between the patients with multiple versus single vascular involvement, between those with versus without a specific other organ involvement such as AIP or between the presence or absence of any of the specific risk factors of atherosclerosis.

### Luminal changes after corticosteroid therapy

**Figure 4:**
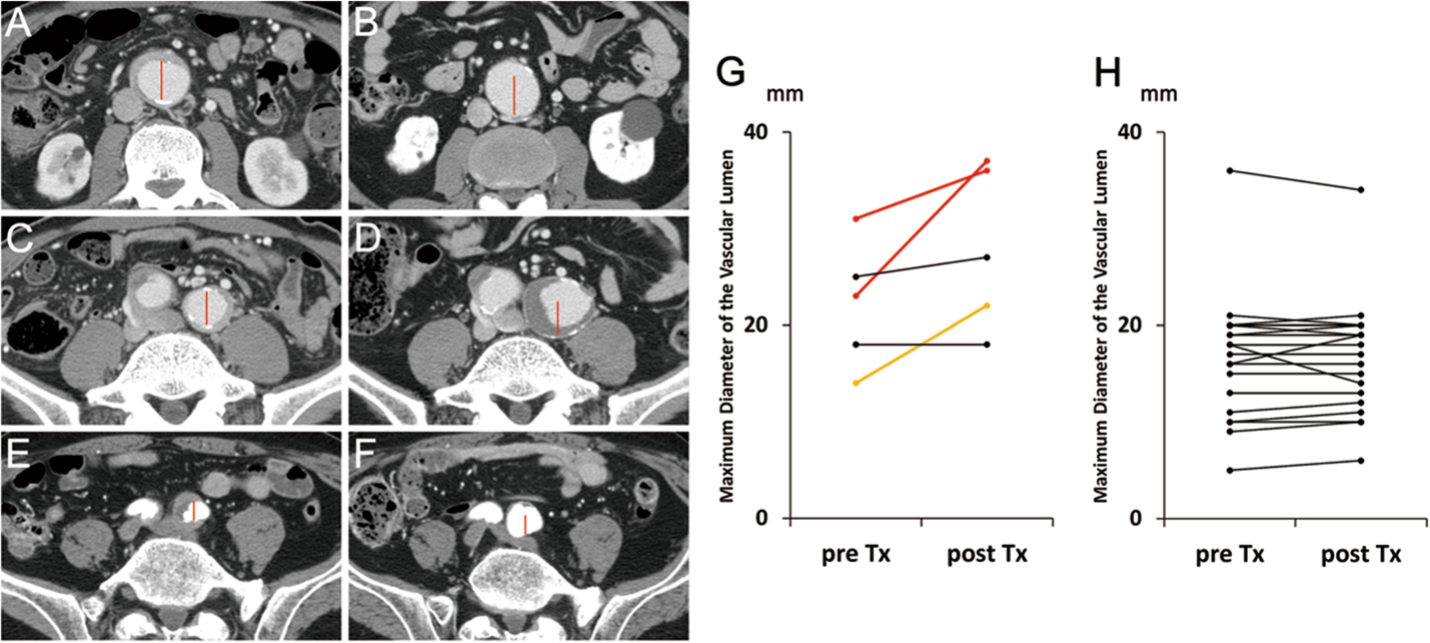
**Exacerbation of luminal dilatation after corticosteroid therapy.** Luminal dilatation (**A** and **C**, patient 10 in Table 1; **E**, patient 9) of the periaortic/periarterial lesions at the time of diagnosis was exacerbated after corticosteroid therapy (**B**, **D** and **F**, respectively). Red lines in each of the three paired images (**A** and **B**, **C** and **D**, and **E** and **F**, respectively) have the same length. Of five lesions from four patients with luminal dilatation, three lesions from two patients (red and yellow) in the maximum luminal diameter increased **(G)**, whereas no obvious increase was observed in the 28 lesions of the 26 patients without luminal dilatation **(H)**. Tx, Treatment.

Of the 31 patients whose clinical course was analyzed after corticosteroid therapy, 5 (patients 4, 9, 10, 18 and 29 in Table 1) had luminal dilatation of the periaortic/periarterial lesions at the time of the initial CT (Figures 4A, 4C and 4E) and three (patients 10, 18 and 29) of them were diagnosed as having inflammatory aneurysm. One (patient 18) of them was treated with EVAR and PSL administration and did not have exacerbation of the luminal dilatation during the follow-up. The other four patients received PSL administration alone, in two (patients 9 and 10) of whom (50%) the luminal dilatation was exacerbated 28 and 46 months after the start of therapy, respectively (Figures 4B, 4D, 4F and 4G). Throughout the clinical course, patient 9 did not have hypertension and patient 10 received antihypertensive agents, which achieved good blood pressure control (below 140/90 mmHg). The luminal diameter was stable after corticosteroid therapy in the 26 patients without luminal dilatation at diagnosis (Figure 4H).

### Outcome of patients without corticosteroid therapy

Four patients (patients 12, 20, 33 and 37 in Table 1) did not receive corticosteroid therapy. One patient (patient 12) treated with antituberculosis therapy had gradual improvement of serum IgG4 level, periaortic/periarterial lesions and retroperitoneal fibrosis. In another untreated patient (patient 20), periaortic/periarterial lesion showed no change during the 24-month follow-up period. No new appearance of luminal dilatation was observed in these two patients. Because follow-up imaging data of the other patients were lacking, we excluded them from the analysis of the clinical course.

## Discussion

We analyzed the clinical course after corticosteroid therapy in patients with IgG4-related PAo/PA. To our knowledge, this study is the largest to evaluate corticosteroid safety and effectiveness in preventing new aneurysm formation in patients without luminal dilatation of periaortic/periarterial lesions, as well as the risk for exacerbation of luminal dilatation of such lesions in patients with it before therapy.

Biopsy of periaortic/periarterial lesions may cause massive hemorrhage. In our study, we could not perform histopathological examinations of these lesions, with the single exception of patient 37, whose specimens obtained by incisional biopsy of periaortic mass lesions showed only findings compatible with IgG4-related retroperitoneal fibrosis because of a lack of vasculature structures. To compensate for this difficulty, the presence of IgG4-related extravascular lesions, in addition to serological and typical radiological findings, was helpful in making a diagnosis of IgG4-related PAo/PA.

In contrast to our present study, the frequency of extravascular lesions was low in several previous studies. In those studies, periaortic/periarterial lesions were at an advanced stage with frequent aneurysmal formation, and the diagnosis was based mainly on the histopathological findings of the periaortic/periarterial lesions themselves because surgical treatment was selected [4, 6, 7, 18, 19]. In contrast, our cases seem to have been at an earlier stage, attributable to the fact that the identification of extravascular lesions and the recent greater awareness of these lesions facilitated the making of an early diagnosis.

Accordingly, in vasculature-restricted cases, the correct diagnosis of IgG4-RD is much more difficult to make. To diagnose such patients early, the indications and accuracy of CT-guided biopsy of periaortic/periarterial lesions should be investigated, the risks and benefits of a diagnostic trial of corticosteroid therapy should be evaluated and a search for other valuable and less-invasive diagnostic markers should be undertaken. In such cases, it is of great importance to exclude other differential diagnoses, such as malignancy, infections, autoimmune disease and drug reactions, which can mimic IgG4-related PAo/PA [20, 21].

A definitive therapeutic strategy for IgG4-related PAo/PA has not been established, and to date indications for treatment and the type of treatment regimen have been decided by the respective attending physician. In type 1 AIP (IgG4-related pancreatitis), the pancreatic manifestation of IgG4-RD, consensus guidelines for treatment, which are based on copious clinical experience [22], have been available since 2010 [23]. In AIP patients, corticosteroid administration should be employed for patients with symptoms such as obstructive jaundice and abdominal and back pain. The initial oral PSL dose of 0.6 mg/kg/day, continuation of the initial dose for 2 to 4 weeks and tapering by 5 mg every 1 to 2 weeks to a maintenance dose (2.5 to 5 mg/day) over a period of 2 to 3 months are recommended. In our study, corticosteroid therapy was started at an initial PSL dose of less than 30 mg/day for eight patients, 30 to 40 mg/day for twenty-four patients and over 40 mg/day for four patients. In most of the patients whose follow-up period was more than 12 months, the PSL dose was tapered to 5 to 10 mg/day by 12 months. In this way, the initial therapy generally following the guidelines of AIP and maintenance therapy with relatively slow tapering were performed in our study, with good efficacy attained on the whole.

The results of this study suggest that luminal dilatation of affected lesions may actually occur during corticosteroid therapy in patients with IgG4-related PAo/PA. In past studies [5, 8, 10], it was speculated that corticosteroid and other immunosuppressive therapies might increase the risk of aneurysm rupture. Actually, an IgG4-RD patient with multiple aneurysms who died of aneurysm rupture after high-dose corticosteroid therapy has been reported [24]. In our present study, of 31 patients treated with corticosteroid, exacerbation of luminal dilatation were observed in only 2 who had already had it before therapy. Because blood pressure was maintained below 140/90 mmHg in these patients throughout their clinical course, hemodynamics seemed not to have influenced the exacerbation of luminal dilatation in any obvious fashion. In contrast, no patient without luminal dilatation showed a new appearance of it after therapy. These results suggest that more careful observation during corticosteroid therapy may be necessary to detect further luminal dilatation early in IgG4-related PAo/PA patients with preexisting luminal dilatation. However, because no patient with luminal dilatation and only one patient without luminal dilatation were observed without corticosteroid therapy, the natural course of the disease or of preexisting dilatation was not clarified. Moreover, the small number of patients with luminal dilatation precluded statistical analysis of the influence of independent risk factors on the luminal dilatation in IgG4-related PAo/PA. Therefore, whether preexisting luminal dilatation, corticosteroid therapy or some other factor is an independent risk factor for the aneurysm formation or exacerbation in IgG4-related PAo/PA patients will have to be clarified through multivariate analysis in a larger prospective study.

This study’s results appear to support the contention that corticosteroid therapy can prevent new appearance of luminal dilatation in patients without it before therapy. In two case reports of IgG4-related aortitis/periaortitis patients with ruptured aortic aneurysms [25, 26], immunosuppressive agents, including corticosteroids, had not been administered before aneurysm rupture. These case reports suggest that IgG4-related PAo/PA is itself a risk for aneurysm formation resulting in rupture when the lesions are left untreated. However, considering that no patient without luminal dilatation showed new appearance of it after therapy in our study, it is reasonable to surmise that corticosteroid therapy improves periaortic/periarterial lesions and prevents aneurysm formation at the affected site.

Therapeutic alternatives to corticosteroids have not been well-established in IgG4-RD. In some case reports and small case series, some oral immunosuppressive drugs, including azathioprine [27], methotrexate [28] and mycophenolate mofetil [29], have been reported to be effective. In addition, good effectiveness of rituximab, which eliminates B cells by binding the cell-surface marker CD20, has been described [30, 31]. However, the efficacy of these drugs remains to be evaluated with regard to their effectiveness for periaortic/periarterial lesions and their influence on luminal dilatation in IgG4-related PAo/PA.

This study has a few limitations. First, the treatment regimen and follow-up protocols were inconsistent between patients because of its retrospective and multi-institutional nature. Second, although this study included more patients than past ones, the number of patients with luminal dilatation at the time of diagnosis was small. Third, the association between histopathological findings and clinical features could not be evaluated, because biopsy specimens for histopathological analysis of the periaortic/periarterial lesions could not be procured. Fourth, no patient with luminal dilatation at the time of diagnosis was observed without corticosteroid therapy.

## Conclusions

The results of our study show the possibility of latent existence and progression of periaortic/periarterial lesions, the efficacy of corticosteroid therapy in preventing new aneurysm formation in patients without luminal dilatation of periaortic/periarterial lesions, and the possibility that a small proportion of patients may actually experience luminal dilatation of periaortic/periarterial lesions in IgG4-related PAo/PA. To confirm the efficacy and safety of corticosteroid therapy in patients with versus without luminal dilatation, and to devise a more useful and safe treatment strategy, including administration of other immunosuppressants, a larger-scale prospective study is required.

## Abbreviations

AIP: Autoimmune pancreatitis
CDC: Comprehensive diagnostic criteria
CRP: C-reactive protein
CT: Computed tomography
EVAR: Endovascular aortic repair
FDG-PET/CT: 2-[^18^F]-fluoro-2-deoxy-D-glucose positron emission tomography/computed tomography
IgG4: Immunoglobulin G4
IgG4-RD: Immunoglobulin G4 (IgG4)–related disease
IgG4-related PAo/PA: IgG4-related aortitis/periaortitis and periarteritis
PSL: Prednisolone.
